# Real-time emulation of parallel channel responses in the vertebrate retina and the primary visual cortex

**DOI:** 10.1186/1471-2202-12-S1-P378

**Published:** 2011-07-18

**Authors:** Hirotsugu Okuno, Tadashi Sanada, Jun Hasegawa, Tetsuya Yagi

**Affiliations:** 1 Graduate school of engineering, Osaka University, Osaka, 565-0871, Japan; 2 Commuture Information Systems Co., Ltd., Osaka, 564-0052, Japan

## 

In order to examine the functional roles of visual neurons in the retina and the cortex, responses of neurons to visual stimuli have been investigated physiologically, and discussions have been made using models derived from the physiological experiments. However, these discussions do not necessarily applicable to the case of highly complex visual environments wherein actual biological vision systems operate since they are based on the responses of a limited number of neurons to simple visual stimuli. In order to examine and discuss the functional roles of the neurons, a system that can emulate the responses of a group of model neurons in real time is required.

Thus far, a high-speed neural computation emulator with transient response has been developed mainly for engineering applications [1]. In this study, we have developed a system for the real-time reconstruction of neural activities with physiologically reasonable spatiotemporal properties using an analog-digital hybrid system comprised of analog resistive networks, field-programmable gate arrays (FPGA), and a digital computer. The system reconstructs activities of 128 x 128 neurons in each neural layer of the retina and the primary visual cortex at 200 frames per second.

**Figure 1:**
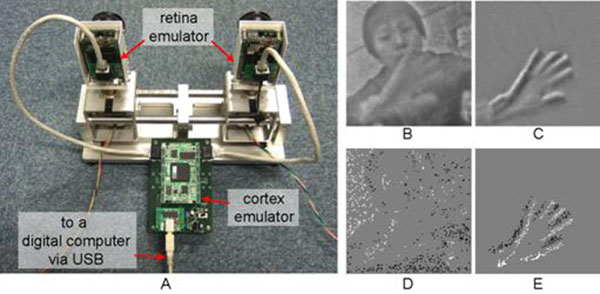
**A.** Appearance of the system comprised of two retina emulators, a cortex emulator, and a digital computer. **B.** Sustained response. **C.** Transient response. **D.** Sustained response with spike representation. **E**. Transient response with spike representation.

The system consists of two retina emulators, a cortex emulator, and a digital computer (Fig.1(A)). The retina emulator emulates both the sustained and transient responses (Fig.1(B)1(C)), and their spike representations (Fig.1(D)1(E)). A part of detailed operation of the retina emulator is written in [2]. The cortex emulator receives spike signals from two retina emulators and emulates responses of cortex neurons including the simple and complex cells.
